# What are the respiratory effects of e-cigarettes?

**DOI:** 10.1136/bmj.l5275

**Published:** 2019-09-30

**Authors:** Jeffrey E Gotts, Sven-Eric Jordt, Rob McConnell, Robert Tarran

**Affiliations:** 1 Department of Medicine, University of California San Francisco, San Francisco, CA, USA; 2 Department of Anesthesiology, Duke University, Durham, NC, USA; 3 Yale Center for the Study of Tobacco Products and Addiction, Department of Psychiatry, Yale School of Medicine, New Haven, CT, USA; 4 Department of Preventive Medicine, University of Southern California, CA, USA; 5 Marsico Lung Institute, The University of North Carolina at Chapel Hill, Chapel Hill, NC 27599 USA; 6 Department of Cell Biology and Physiology, The University of North Carolina at Chapel Hill, Chapel Hill, NC 27599 USA

## Abstract

Electronic cigarettes (e-cigarettes) are alternative, non-combustible tobacco products that generate an inhalable aerosol containing nicotine, flavors, propylene glycol, and vegetable glycerin. Vaping is now a multibillion dollar industry that appeals to current smokers, former smokers, and young people who have never smoked. E-cigarettes reached the market without either extensive preclinical toxicology testing or long term safety trials that would be required of conventional therapeutics or medical devices. Their effectiveness as a smoking cessation intervention, their impact at a population level, and whether they are less harmful than combustible tobacco products are highly controversial. Here, we review the evidence on the effects of e-cigarettes on respiratory health. Studies show measurable adverse biologic effects on organ and cellular health in humans, in animals, and in vitro. The effects of e-cigarettes have similarities to and important differences from those of cigarettes. Decades of chronic smoking are needed for development of lung diseases such as lung cancer or chronic obstructive pulmonary disease, so the population effects of e-cigarette use may not be apparent until the middle of this century. We conclude that current knowledge of these effects is insufficient to determine whether the respiratory health effects of e-cigarette are less than those of combustible tobacco products.

## Introduction

The lungs are a physiologic marvel, transmitting the entire cardiac output through around 2000 km of capillaries with each heartbeat and performing gas exchange in 300 000 000 alveoli with a surface area of about 70 m^2^. With every breath, this highly adapted and delicate organ is exposed to infectious and inflammatory environmental stimuli. As a result of innate and acquired immunity, inspired air is cleaned and humidified before it reaches the alveoli. However, a failure of these processes leads to infection, inflammation, lung damage, and impaired gas exchange.

In considering the effects of electronic cigarettes (e-cigarettes) on lung health, we begin with a brief history of traditional cigarettes. Cigarette smoking accelerated in the late 19th and early 20th centuries in tandem with the growth of mass production technologies and advertising.^1^ However, it was not until the 1930s that statisticians noted increased cancer mortality rates and thoracic surgeons reported an increase in pneumonectomy to remove lung cancers.^2^ Three decades later the landmark 1964 US Surgeon General’s Report causally attributed lung cancer to cigarette smoking,^3^ and four decades after this the tobacco companies were defeated in the US court system on racketeering charges that they systematically deceived the public in the pursuit of profits. The lesson from smoking in the 20th “cigarette” century is that it took decades to show that addictive, heavily marketed inhaled tobacco products caused lung disease. It is therefore imperative that the scientific community uses all available modalities to define the health effects of novel tobacco products so that public health policy can be based on evidence.

E-cigarettes use a metal resistance coil to heat and aerosolize mixtures of vegetable glycerin, propylene glycol, nicotine, and flavoring agents. E-liquids are conducted from a tank to the coil by a wick made of cotton, silica, or ceramic, and the user activates electric current through the coil by depressing a button or by generating airflow through the device. Since their introduction 15 years ago, e-cigarettes have undergone major changes in design that allow the user greater control over e-liquid composition, nicotine concentration, and how the e-liquid is aerosolized/vaped.

The health effects of exposure to e-cigarettes, especially of chronic exposure, are uncertain. However, e-cigarettes emit volatile carbonyls, reactive oxygen species, furans, and metals (nickel, lead, chromium),^4^ many of which are toxic to the lung. This review summarizes the rapidly expanding evidence from cell culture, animal, and human studies on the potential pulmonary health effects of e-cigarettes.

## Sources and selection criteria

We identified references for this review through searches of publications listed by PubMed from 1980 to 30 June 2019. Owing to the recent reports of severe lung injury associated with e-cigarette use, we also did an additional search in September 2019 and subsequently included several additional relevant studies published in September 2019, which were added during the proofing stage. We used the search terms “e-cigarette”, “vape”, “juul”, “lung”, “airway”, “respiratory”, “cough”, “methacholine”, “nasal”, “alveolar”, “alveoli”, “immune”, “bronchial, “tracheal”, “bronchoalveolar”, “nicotine”, “propylene glycol”, “vegetable glycerin”, “neutrophil”, “macrophage”, “epithelia”, “spirometry”, and “FEV_1_”. We also identified references from relevant review articles. We included in vitro, animal, and human studies, including meta-analyses. Only articles published in English were reviewed. We screened more than 5000 articles of evidence classes I-IV and included classes I-III. We excluded articles published in non-peer reviewed journals and small uncontrolled series, with the exception of case reports of human lung disease associated with e-cigarette use. The final reference list was based on relevance to the topics covered in the review.

The tobacco industry has a long history of conducting studies intended to create doubt about the health effects of combustible tobacco and of misinterpreting data, and as tobacco companies consolidate their control of the $11.4bn (£9.3bn; €10.3) global e-cigarette market (projected to reach $86.4bn in 2025),^5^ traditional industry marketing, lobbying, and research strategies are increasingly apparent. Moreover, industry funding is strongly associated with finding no harm of e-cigarettes, compared with studies without a potential conflict of interest (odds ratio 67, 95% confidence interval 8 to 553).^6^ We have included studies funded by the tobacco industry in our review, but we have identified them as such.

## Rates of e-cigarette use

More than a billion people worldwide smoked tobacco in 2016. In the US, 34.3 million (14.0%) adults (≥18 years of age) were current smokers in 2017; 6.9 million (2.8%) were current e-cigarette users.^7^ ^8^ Rates of e-cigarette use are higher in young people and have accelerated recently.^9^ Among 8th, 10th, and 12th grade pupils in the Monitoring the Future study, for example, prevalence rates of vaping (e-cigarette use) in the US in 2018 were 9.7%, 20%, and 25%, respectively.^10^ ^11^ Increases in 2018 in 10th and 12th grade pupils were the largest recorded for any substance in the 44 years that the study has tracked adolescent drug use.

## Device evolution and the rise of Juul

E-cigarettes have undergone dramatic changes in design to deliver nicotine more efficiently.^12^ ^13^ ^14^ ^15^ Initially, most sales came from “ciga-like” products that resembled traditional cigarettes.^16^ Modular systems (mods), containing batteries, fillable liquid tanks, and heating coils have a comparably small but loyal following. They allow the use of custom-made flavors and individualized settings for temperature and wattages.^14^

Newer pod devices, beginning with the Juul e-cigarette system, have seen a rapid rise in use in the US. Introduced in mid-2015, Juul had a dominant market position by 2018, accounting for more than 70% of US e-cigarette sales.^16^ ^17^ ^18^ During this time, overall US sales of e-cigarettes doubled, with Juul being responsible for the bulk of market growth. The Juul device resembles a USB memory stick with cartridges (pods) that are exchangeable by the user and filled with flavored e-liquids. Juul e-liquids contain nicotine in protonated form, generated by titration with benzoic acid to yield the nicotine benzoate salt. Users perceive aerosols produced from liquids containing nicotine salt as less irritating, allowing delivery of higher amounts of nicotine than in aerosols from traditional e-liquids that contain freebase nicotine. The nicotine concentration in US marketed Juul liquid cartridges (pods) is higher (5% weight/weight; 59 mg/mL; 127 mM) than in traditional e-liquids (typically 6-18 mg/mL of nicotine; 37-111 mM). Recent studies have shown that Juul users have similar blood nicotine concentration profiles to users of combustible cigarettes.^19^

## Human studies

### Studying lung toxicity

**Fig 1:**
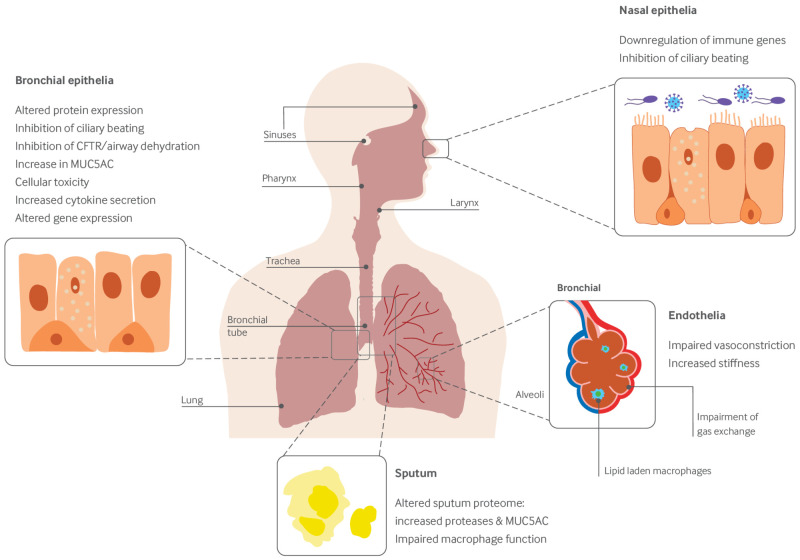
Reported effects of vaping on the human pulmonary system

The respiratory system varies dramatically in its composition and function from the large airways to the alveolar space. Proximally, the airways function to conduct air to the deeper lung while protecting it from injurious toxicants and microorganisms. To this end, they have a complex structure with cartilaginous elements anteriorly for structural support and a collapsing posterior wall to enable high airspeed velocity during coughing, nervous system innervation, a smooth muscle layer to facilitate bronchoconstriction, glands and surface epithelia that produce a mucous layer that hydrates the underlying epithelium and traps microbes, cilia that transport mucus away from the alveolar space, and extensive lymphatics. In contrast, the alveoli are delicate structures lined by thin alveolar type 1 epithelial cells and surfactant producing alveolar type 2 cells, along with alveolar macrophages. A single fused basement membrane separates the alveolar epithelium and capillary endothelium, yielding a remarkably thin alveolar-capillary barrier of approximately 5 µm to facilitate gas diffusion.^20^ Given the vast differences between these two regions, toxicological investigations should focus on both the conducting airways and the alveolar spaces. Figure 1 shows the reported effects of vaping on the human pulmonary system.

### Population studies

Users of e-cigarettes have reported several negative symptoms involving the nose, mouth, throat, and airways.^21^ However, few epidemiologic studies have looked at chronic effects of e-cigarette use in either young people starting e-cigarettes or smokers transitioning to exclusive e-cigarette use.

Surveys of adolescents have found increased risk of respiratory symptoms. A survey of approximately 45 000 adolescents in Hong Kong found that e-cigarette use in the previous month was associated with increased odds of reporting chronic cough or phlegm (odds ratio 2.1, 95% confidence interval 1.8 to 2.5).^22^ A study of almost 2000 high school students in Southern California, of whom nearly 10% were current (previous 30 days) e-cigarette users, reported that both past and current use were associated with a nearly twofold increase in the risk of chronic bronchitic symptoms (chronic cough, phlegm, or bronchitis), a finding that was robust to adjustments for sociodemographic confounders and cigarette use and was also observed in a sensitivity analysis restricted to those who had never used cigarettes.^23^ A large survey of Korean high school students found that self reported diagnosis of asthma by a physician in the previous year was increased in current e-cigarette users compared with never users, after adjustment for exposure to cigarette smoke (odds ratio 2.7, 1.3 to 5.8).^24^

Other cross sectional surveys have also found associations of e-cigarette use with a history of asthma and with asthma exacerbations on the basis of state-wide surveys of young people in Hawaii and Florida.^25^ ^26^ ^27^ In adults, a recent analysis of data from the Behavioral Risk Factor Surveillance Survey found an association of e-cigarette use with asthma or chronic obstructive pulmonary disease (COPD), effects that were larger in non-smokers.^28^ In a population based study in Sweden, associations of e-cigarette use with respiratory symptoms (chronic cough, sputum, or wheeze) were strongest among dual users with cigarettes.^29^ Associations among non-smokers and never smokers were weaker and not statistically significant. Finally, a recent study of nearly 40 000 participants in the Health eHeart Study found that e-cigarette use was associated with higher self ratings of dyspnea and reports of COPD and asthma.^30^ These studies were cross sectional, and outcomes were self reported. Prospective cohort studies are needed, but the consistency of these associations among both young and adult e-cigarette users suggest that e-cigarette users experience symptoms of both airway and alveolar injury, which are consistent with the studies of human and animal lungs, as discussed below.

In the summer of 2019, several hundred cases of acute respiratory illness associated with e-cigarette use were reported in the US, prompting multiple investigations by state and federal health agencies, including the Centers for Disease Control and Prevention (CDC). As of writing, seven deaths have been attributed to e-cigarette use, and investigations are ongoing.^31^ The clinical presentation of 53 affected vapers in Illinois and Wisconsin was recently described.^32^ Presenting symptoms included gastrointestinal (81%), constitutional (100%), and respiratory (98%) symptoms, with 87% of patients reporting dyspnea and 83% reporting cough. Most patients had arterial hypoxemia (69%), elevated blood neutrophil counts (94%), and elevated transaminases (55%). Fourteen patients underwent bronchoscopy with cellular analysis, showing an elevated median neutrophil percentage (65%) and corresponding reduction in macrophage percentage (median 21%). Seven bronchoalveolar lavage samples were stained with oil red O stain and showed lipid laden macrophages. Fifteen patients were diagnosed as having acute respiratory distress syndrome. and most patients showed abnormal chest radiography. All 48 patients scanned by computed tomography were found to have abnormal lung parenchyma, typically characterized by ground glass opacities in both lungs, sometimes with subpleural sparing. These findings have been monitored by the CDC, and similar findings have been reported in 25 states.^33^ Whether this novel “vaping associated respiratory syndrome” is caused by propylene glycol/vegetable glycerin and nicotine containing e-cigarettes, or is due to tetrahydrocannabinols and/or associated solvents and adulterants such as vitamin E, remains to be determined.^34^ However, similar cases have been found in the UK and Japan, suggesting that this has the potential to be a more widespread phenomenon, although the country to country variations in frequency remain to be determined.^35^ ^36^

### Studies of smokers who switch to e-cigarettes

Several groups have studied symptom scores and spirometry among chronic smokers who transition to e-cigarette use. This is a useful design for assessing the respiratory effects of e-cigarettes; although some studies have found that e-cigarette users experience improvements in lung health, results have not been consistent even when users were able to reduce cigarette consumption.^37^ ^38^ ^39^ ^40^ Studies funded by the tobacco industry have consistently found few adverse respiratory health outcomes in smokers transitioning to e-cigarette use.^41^ ^42^ ^43^ An important caveat to these studies is that in general they have not looked at whether e-cigarettes have respiratory toxicity but at whether the measured outcomes differ from those of people exposed to ongoing cigarette smoke. To answer this question, studies would need to compare smokers who have transitioned to exclusive e-cigarette use with smokers who have quit without any intervention or with nicotine replacement therapy or approved pharmacotherapy, the current gold standard for treatment.

### Studies of spirometry

Spirometry involves forced inhalation and exhalation maneuvers and the monitoring of airflow over time. This test is reproducible under optimal conditions (consistent patient effort with calibrated equipment), allowing assessment of airway obstruction and affording some insight into lung volumes. Importantly, it gives a limited window into lung function because it does not assess for restrictive lung physiology or gas exchange abnormalities and may change rapidly with exercise and other stimuli. For example, transient airway smooth muscle contraction, which can occur during exercise, is detectable during spirometry and can subsequently resolve within minutes to hours. The forced expiratory volume in one second (FEV_1_) is considered to be a measure of air clearance from the large/cartilaginous airways, and reductions in FEV_1_ and/or the ratio of FEV_1_ to forced vital capacity (FEV_1_/FVC) may be due to smooth muscle contraction (as has been seen in animal studies) or may instead be a sign of more significant and long lasting structural lung damage.

Most spirometric studies of e-cigarette users have focused on acute changes in airflow immediately after a vaping session. These studies show mixed results, with some reporting evidence of airflow obstruction and others not.^44^ ^45^ ^46^ ^47^ ^48^ Notably, patients with pre-existing airway disease may be at higher risk of acute airway obstruction with e-cigarette exposure.^49^

Few longer term studies of e-cigarette use and spirometry after a period of abstinence have been performed. One study compared 30 healthy daily users of e-cigarettes (excluding current and former cigarette smokers) with 30 controls.^50^ The groups were matched for age, height, weight, ethnicity, and socioeconomic status; abstinence from vaping was required for at least one hour before testing. E-cigarette users were found to have lower FEV_1_ (4.6 (SD 0.7) L *v* 5.2 (0.8) L; P=0.007) and FEV_1_/FVC (77.4 (7.2) *v* 83.4 (5.6); P=0.001) compared with the control group, but spirometry was performed after a minimum of only one hour of abstinence, thus potentially reflecting acute bronchospasm rather than lasting changes in the airways.^50^ Smokers who have been studied after transitioning to e-cigarettes have been found to have either no change or slight improvements in spirometry.^37^ ^39^ Importantly, the absence of short term changes in spirometry does not mean that e-cigarettes are harmless. Patients with cystic fibrosis, for example, are born with normal lungs but develop lung disease over time, and young (~4 year old) cystic fibrosis patients have normal FEV_1_, even though they have extensive chronic lung disease as measured by lung clearance index or by imaging.^51^ Similarly, changes in spirometry can reliably be detected only after years or decades of exposure to cigarette smoking, despite substantial injury to the distal lung that can be measured by other means.^52^ For example, significant pathologic changes, including small airway/alveolar destruction, have been observed in early stage smoking induced COPD by using imaging techniques, despite relatively mild changes to FEV_1_.^53^

### Airway inflammation and injury

Because of the delicate nature of the lungs, even mild inflammation can be damaging.^54^ Lung inflammation can be assessed histologically, by analysis of bronchoalveolar lavage, or by studying lung homogenates. Healthy e-cigarette users have been found to have erythematous and irritable airway mucosa,^55^ and cases of more serious bronchial injury have been reported.^56^ Increased levels of the MUC5AC mucin have been found both in bronchial epithelia and in airway secretions, although notably many of these e-cigarette users were former smokers.^55^ ^57^ Increased mucin levels inversely correlate with the decline in lung function in COPD patients and are a biomarker of chronic bronchitis, indicating that mucins are a validated biomarker of harm.^58^ ^59^

Although increased exhaled nitric oxide may be suggestive of airway inflammation in asthma, exhaled nitric oxide can be decreased under conditions of high oxidative stress, as in COPD, making its interpretation less clear.^60^ Several groups have reported reductions in the fractional excretion of nitric oxide following exposure to e-cigarette aerosol.^50^ ^61^ Furthermore, proteomics of e-cigarette users’ sputum has shown higher levels of neutrophil activation, including myeloperoxidase, neutrophil elastase, and proteinase-3.^57^ Increased protease concentrations have previously been observed in tobacco smokers’ lungs,^62^ ^63^ ^64^ ^65^ and the association between smoking, increased proteolysis, and lung damage is causal, suggesting that protease concentrations are another biomarker that may be useful for studying the effects of vaping on the lung.^66^ ^67^ ^68^ When dysregulated, lung proteases can degrade basement membranes and lead to emphysema, as seen in COPD patients.^69^ Increased proteolysis also causes bronchiectasis in cystic fibrosis and α1 anti-trypsin deficiency-type lung diseases,^70^ ^71^ and it plays an important role in tumor progression/metastasis by facilitating tissue remodeling.^72^

Finally, a recent controlled study in healthy young occasional smokers and middle aged heavier smokers showed that just 15 minutes of exposure to aerosol from a 60 W e-cigarette impaired gas exchange and reduced expiratory gas flows, in association with increased blood concentrations of the lung specific protein CC16 that is secreted by club cells located near the terminal bronchioles.^73^ These results suggest that e-cigarettes may cause acute, physiologically detectable injury to the small airways.

### Alveolar inflammation and injury

Given that cigarette smokers are at increased risk of life threatening alveolar injury and acute respiratory distress syndrome,^74^ ^75^ prioritizing further studies of possible subclinical alveolar injury from e-cigarettes will be important. A rapidly increasing number of case reports link e-cigarette use to severe inflammatory diseases affecting the small airways and alveoli: lipoid pneumonia,^76^ eosinophilic pneumonia,^77^ diffuse alveolar hemorrhage,^78^ organizing pneumonia,^77^ ^79^ respiratory bronchiolitis associated interstitial lung disease,^80^ and hypersensitivity pneumonitis.^81^

Hypersensitivity pneumonitis is caused by an inflammatory reaction against known inhalational antigens that commonly progresses to life threatening pulmonary fibrosis. One study exposed healthy volunteers with no history of cigarette or e-cigarette use to a single session of vaping. Analysis of blood samples showed increases in endothelial microparticles, which are shed from endothelia, suggesting that alveolar capillaries were activated or injured with this relatively mild exposure.^46^ Beyond this, comparatively little has been done to evaluate the effects of e-cigarette aerosol at the alveolar level in humans. Whether the case reports represent individual susceptibility, effects of the extraordinary diversity of flavors and other molecular products of e-cigarettes, or both is unknown. However, that e-cigarettes pose risks for hypersensitivity pneumonitis and other alveolar diseases at the population level is plausible.

### Effects on immunity

Reporting of respiratory symptoms by e-cigarette users suggests increased susceptibility to and/or delayed recovery from respiratory infections. A study of 30 healthy non-smokers exposed to e-cigarette aerosol found decreased cough sensitivity.^82^ If human ciliary dysfunction is also negatively affected, as suggested by animal and cellular studies,^83^ the combination of reduced coughing and impaired mucociliary clearance may predispose users to increased rates of pneumonia. Exposure to e-cigarettes may also broadly suppress important capacities of the innate immune system. Nasal scrape biopsies from non-smokers, smokers, and vapers showed extensive immunosuppression at the gene level with e-cigarette use.^84^ Healthy non-smokers were exposed to e-cigarette aerosol, and bronchoalveolar lavage was obtained to study alveolar macrophages.^46^ The expression of more than 60 genes was altered in e-cigarette users’ alveolar macrophages two hours after just 20 puffs, including genes involved in inflammation. Neutrophil extracellular trap (NET) formation, or NETosis, is a mode of innate defense whereby neutrophils lyse DNA and release it into the extracellular environment to help to immobilize bacteria, a process that can also injure the lung.^85^ Neutrophils from chronic vapers have been found to have a greater propensity for NET formation than those from cigarette smokers or non-smokers.^57^ Given that e-cigarettes may also impair neutrophil phagocytosis,^86^ these data suggest that neutrophil function may be impaired in e-cigarette users.

## Animal studies

Animal models provide a useful tool for studying the potential effects of exposure to e-cigarettes. The utility of animal models using intense exposure paradigms to shorten timescales and simplify experimental design was originally demonstrated conclusively in studies in mice identifying causal effects and mechanisms of oncogenesis induced by cigarette smoke.^87^ ^88^ ^89^ Approximately 60 such studies of effects of e-cigarettes have been performed in mice, with durations of exposure ranging from a single dose to three to six months using propylene glycol/vegetable glycerin with or without nicotine and commercial e-liquids (table 1). Acute exposures have caused changes at the level of the protein, including up-regulation of mucins and cytokines, as well as cellular changes including impaired autophagy (table 1). Chronic e-cigarette exposures induce airway inflammation, neutrophilia, airway remodeling, and emphysema (table 1). Increased sensitivity to methacholine was also observed (table 1). Both nicotine dependent and nicotine independent effects were found. Although most studies found significant effects of vaping, two industry funded studies did not,^103^ ^105^ consistent with the previously mentioned relation between industry funding and observed effects of vaping.^6^

**Table 1:** Pulmonary effects of vaping in animal models

Observation	Species	Duration of exposure (e-liquid)
Airway hyper-reactivity/bronchospasm^90^-^93^	Mice, guinea pigs	Acute (Old Kentucky e-liquid 12 mg/mL nicotine); 6 weeks, 1 h/day, 5 days/week (American Tobacco e-liquid, PG and VG ±12 mg/mL nicotine); intratracheal e-liquid instillation twice weekly for 10 weeks (Z-company e-liquid 16/mg/mL diluted 50 times in saline); 3 and 28 days (PG/VG or ±18 mg/mL nicotine or American Tobacco e-liquid)
Increase in MUC5AC mucin or goblet cell metaplasia^55^ ^91^ ^94^	Mice, rats	One 3 h exposure (PG/VG only); 3 and 28 days (PG/VG or ±18 mg/mL nicotine or American Tobacco e-liquid); 90 days (PG/VG ±18 mg/mL nicotine)
Reduction in mucus clearance^95^	Mice	1-3 weeks, 20 min/day (PG ±24 mg/mL nicotine)
Increased inflammation^94^ ^96^-^101^	Mice, rats	1 h/day for 4 months (PG/VG ±18-24 mg/mL nicotine); one dose, lungs harvested 0.5 or 24 h later (commercial e-liquids with nicotine); 1.5 h, twice daily for 2 weeks (NJOY menthol bold, 1.8% nicotine); 3 days, 5 h/day (Blu e-cig, classic tobacco 16 mg/mL nicotine); 1 h/day, 5 days/week, for 4 weeks (6-24 mg/mL commercial e-liquids); 90 days (PG/VG ±18 mg/mL nicotine)
Increased PAFR expression^88^ ^98^	Mice	Twice daily intranasal dosing with e-liquid for 4 days (24 mg/mL nicotine containing e-liquid)
Nicotine dependent weight loss^90^ ^93^	Mice, rats	6 weeks, 1 h/day 5 days/week (American Tobacco e-liquid, PG and VG ±12 mg/mL nicotine); intratracheal e-liquid instillation twice weekly for 10 weeks (Z-company e-liquid 16/mg/mL diluted 50 times in saline)
Impaired bacterial clearance and/or altered virulence^97^-^99^	Mice	1 h/day, 5 days/week, for 4 weeks (6-24 mg/mL commercial e-liquids); 1.5 h, twice daily for 2 weeks (NJOY menthol bold, 1.8% nicotine)
Oxidative stress^97^ ^100^	Mice	1.5 h, twice daily for 2 weeks (NJOY menthol bold, 1.8% nicotine); 3 days, 5 h/day (Blu e-cig, classic tobacco 16 mg/mL nicotine)
Impaired autophagy, increased aggresome formation^102^	Mice	3 × 1 h exposure in 24 h (PG/VG and 25 mg/mL nicotine)
Nasal squamous cell metaplasia^103^ *	Rats	90 days, 6 h/day, 5 days/week (PG/VG ± ≤5 mg/mL nicotine)
DNA adducts found in lung, bladder and heart; extrapulmonary effects^98^ ^104^	Mice	1 h/day, 5 days/week, 3-6 months (PG/VG ±24 mg/mL nicotine)
Emphysema^96^	Mice	1 h/day for 4 months (PG/VG ±18 mg/mL nicotine)
Limited or no pulmonary effects^103^ ^105^ *	Mice, rats	4 h/day for 1-3 weeks (MarkTen 250 μM nicotine); 90 days, 6 h/day, 5 days/week (PG/VG ± ≤5 mg/mL nicotine)

PAFR=platelet activating factor; PG=propylene glycol; VG=vegetable glycerin.

* Studies funded by tobacco industry.

Thus, vaping in mice leads to rapid changes at the cell and protein levels. Of greater concern, five to 16 weeks of e-cigarette exposure induced alveolar cell apoptosis and architectural simplification suggestive of emphysema,^96^ ^106^ although results have not been consistent.^90^ ^107^ Finally, although e-cigarettes have been widely promoted as having a negligible risk of malignancy compared with smoked tobacco, exposure to e-cigarette aerosol has recently been associated with DNA damage, thought to occur via in situ metabolism of nicotine to nitrosamines.^91^

Two weeks of exposure to e-cigarette aerosol in mice decreased survival and increased pathogen load following inoculation with either *Streptococcus pneumoniae* or influenza A, two leading causes of pneumonia in humans.^97^ Furthermore, the aerosol exposure may lead to enhanced upper airway colonization with pathogens and to virulent changes in pathogen phenotype, as shown with *Staphylococcus aureus*.^98^ ^99^ Thus, although more studies are needed, the animal data suggesting that vaping leads to an increased susceptibility to infection would seem to correlate with the population level data in young adult humans, whereby vapers have increased rates of symptoms of chronic bronchitis.^23^

## In vitro studies of vaping

### Methodological considerations

Different exposure paradigms have been used to study e-cigarettes. Direct addition of e-liquids to in vitro systems does not replicate real world vaping. However, given that thousands of e-liquids are commercially available, it yields increased throughput. Condensing vaped e-liquids is an intermediate approach, although condensate can change over time and the effects of short lived reactive products may be missed. Direct exposure to e-liquid aerosol may be more relevant. However, unlike cigarette puff topographies that are well studied and for which standard dosing approaches have been recommended,^108^ e-cigarette topographies are not only poorly understood but are changing as new e-cigarette devices emerge. Owing to the lack of standards, dosimetry should be done to verify that aerosol is produced and reaching its target cells. E-liquids/vaping have been studied in vitro using a variety of cell culture systems and exposure models. Although immortalized cellular systems are useful for studying large numbers of e-liquids, we focus on vaping effects on primary pulmonary cells, as they have greater in vivo relevance.

### Airway epithelia

Airway epithelia play important roles in sterilizing and humidifying inhaled air. They secrete ions/water, mucins, and cytokines and clear mucus via ciliary beating.^100^ Exposure to tobacco smoke decreases ciliary beat frequency both in smokers and in vitro^109^ ^110^ ^111^ ^112^ Ion transport is also impaired and mucin/cytokine secretion is elevated both in smokers and in vitro.^101^ Therefore, these cellular biomarkers of harm may be applicable to vaping studies. Acute exposure to e-cigarette vapor has been shown to rapidly decrease ciliary beating, inhibit mitochondrial function, and alter the expression of genes involved in oxidative and xenobiotic stress pathways (table 2),^83^ ^115^ ^121^ mirroring changes at the protein level in bronchial epithelia obtained by bronchoscopy from vapers.^55^ Genes involved in ciliogenesis were also altered, which is consistent with the functional data showing impaired ciliary beating after vape exposure.^83^ ^115^

**Table 2:** Summary of effects of vaping on primary pulmonary cells in vitro

Observed effect	Cell type	E-liquids	Delivery methods
Airway surface dehydration and/or inhibition of CFTR ion transport^96^ ^113^ ^114^	Human bronchial epithelia	Red oak e-liquid, 1% nicotine; e-liquid ±36 mg/mL nicotine	Aerosol delivery to cultures
Decreased ciliary beating^83^ ^96^ ^114^ ^115^	Human bronchial and nasal epithelia	e-liquid ±36 mg/mL nicotine; e-liquid ±36 mg/mL nicotine	Aerosol delivery to cultures
Increased MUC5AC mucin production^55^	Human bronchial epithelia	PG/VG (nicotine independent)	Aerosol delivery to cultures
Decreased cell viability/increased cellular toxicity^86^ ^115^-^117^	Human bronchial epithelia, alveolar macrophages, airway smooth muscle, NK cells	Several commercial e-liquids, all at 12 mg/mL nicotine; vaped e-liquid condensate and/or aerosol from second and third generation devices	Liquid and aerosol delivery to cultures
Increased cytokine secretion^86^ ^118^	Human alveolar macrophages, bronchial epithelia, peripheral blood neutrophils, NK cells	Several flavored nicotine-free e-liquids; commercial e-liquids ±24 mg/mL nicotine	Vaped e-liquid condensate from third generation device
Altered membrane fluidity^55^	Human bronchial epithelia	PG/VG (nicotine independent)	Aerosol and liquid delivery to cultures
Decrease in barrier function (resistance)^119^ ^120^	Human, mouse, and rat endothelia; human/COPD bronchial epithelia	Commercial e-liquids; up to 25 mM nicotine; USA tobacco flavor, 24 mg/mL nicotine	Condensate generated
Impaired phagocytosis^86^ ^116^	Human alveolar macrophages and peripheral blood neutrophils	Several flavored nicotine-free e-liquids; commercial e-liquids ±24 mg/mL nicotine	Vaped e-liquid from third generation device
Impaired mitochondrial function and reduced glycolysis^115^	Human bronchial epithelia	Cinnamaldehyde flavored e-liquid	Vaped e-liquid directly and as condensate from third generation device
p38 MAPK and/or ERK activation^96^ ^118^ ^119^	Human bronchial epithelia; human, mouse, and rat endothelia; human neutrophils	e-liquid ±36 mg/mL nicotine; commercial e-liquids; up to 25 mM nicotine; commercial e-liquids ±24 mg/mL nicotine	Aerosol delivery to cultures; condensate generated using second or third generation devices
Induction of apoptosis and necrosis^116^	Human alveolar macrophages	Commercial e-liquids ±36 mg/mL nicotine	Condensate generated using second generation device
Changes in gene expression^121^	Human bronchial epithelia	VitroCell System	Increased genes involved in oxidative and xenobiotic stress markers of ROS; decreased genes involved in ciliary function
No change in barrier function (resistance), CBF, FOXJ1, MUCAC; essentially no change in RNA transcript expression^122^ *	Human bronchial epithelia	“Blended tobacco” e-liquid 18 mg/mL nicotine	Vype E-pen; cells exposed to vapor in BAT exposure chambers
No effect on ASL height, ion transport or CBF^123^ *	Human bronchial epithelia	Avail Vapor “Tobacco Row” 18 mg/mL nicotine	Aerosol generated using third generation device

ASL=airway surface liquid; BAT=British American Tobacco; CBF=ciliary beat frequency; COPD=chronic obstructive pulmonary disease; CSTR=cystic fibrosis transmembrane conductance regulator; NK=natural killer; PG=propylene glycol; ROS=reactive oxygen species; VG=vegetable glycerin.

* Studies funded by tobacco industry.

Cystic fibrosis transmembrane conductance regulator (CFTR) is an apical membrane anion channel expressed in airway epithelia. Lack of functional CFTR causes cystic fibrosis lung disease. Acute and chronic cigarette smoke exposures rapidly inhibit CFTR function in smokers and in vitro.^124^ ^125^ ^126^ Similarly, e-cigarettes can also inhibit CFTR mediated Cl^–^ secretion and induce airway dehydration in airway epithelia.^113^ Failure of ciliary beating induced by e-cigarette aerosol could conflate this, contributing to a retention of mucus and bacteria, increasing the chance of developing lung disease (table 2). Whether vaping inhibits CFTR in vivo has yet to be ascertained; however, as CFTR function can be assessed using minimally invasive electrophysiologic approaches, measuring CFTR function may be a useful biomarker of harm that can be tested in the vaping population.

### Immune cells

E-cigarette condensate induces alveolar macrophage apoptosis, increases pro-inflammatory cytokine secretion, and impairs phagocytosis.^116^ Similarly, direct e-liquid addition has been shown to impair macrophage phagocytosis.^86^ Neutrophils exposed to e-liquids have impaired phagocytosis, increased cytokine secretion, and increased NET release.^86^ Aerosol extract has been shown to cause morphologic changes in neutrophils, alter the expression of pro-inflammatory surface markers CD11b and CD66b, and increase the release of proteases and inflammatory cytokines.^118^ Secretion of these proteins, identified in the sputum of chronic e-cigarette users,^57^ would be predicted to increase neutrophil recruitment to the lung and increase degradation of collagen that could facilitate airway remodeling, lead to lung damage, or both. Importantly, whether the effects of e-cigarettes on neutrophils would be seen only in the lung or would extend to neutrophils in the pulmonary or extra-pulmonary blood supplies is not known. Certainly, although more studies on the effects of vaping on immune cells are needed, the in vitro studies seem to show that vaping can both activate immune cells and impair some of their key functions.

### Endothelia

Studies have assessed the effects of e-cigarette exposure on the lung’s microvasculature. E-cigarettes decreased the electrical resistance of endothelial cells derived from mice, rats, and humans, as well as exerting significant effects on cell viability that were associated with changes in cell signaling (activation of p38 MAPK). These changes are similar to those observed after exposure to cigarette smoke extract.^119^

### Prokaryotes

The airways are constantly exposed to both inhaled and oral bacteria. However, although the normal upper airways and oral cavity have measurable microbiomes, the distal airways are typically sterile owing to the ability to clear inhaled or aspirated bacteria. In cystic fibrosis and COPD, a lower airways microbiome develops.^127^ ^128^ Few studies have looked at the effects of vaping on bacteria relevant to the lung, and none has investigated vapers’ lung microbiomes. However, acute vaping in humans increased expression of platelet activating factor receptor, a receptor expressed in airway epithelia.^98^ Crucially, this receptor enables *Streptococcus pneumoniae* to adhere to mammalian cells, and, in vitro, vaping increased both platelet activating factor receptor expression and adherence of *S pneumoniae* to airway epithelia. Similarly, chronic vaping was found to increase the virulence of *Staphylococcus aureus* and lead to increased biofilm formation. With chronic exposure to tobacco smoke, years are needed to alter the lower airways microbiome. Thus, a monitoring of vapers’ lungs over a similar timeframe will likely be needed.

## Toxicity of specific aerosol components

### Propylene glycol and vegetable glycerin

As well as being a base constituent in e-liquids, propylene glycol is a common chemical used to produce polyester and as de-icer/antifreeze. Intravenous propylene glycol can cause acute renal and central nervous system toxicity.^129^ Propylene glycol has previously been shown to inhibit renal glucose transport and corneal Na^+^/K^+^ATPase activity.^130^ ^131^ Propylene glycol and vegetable glycerin are classified as “generally recognized as safe,” if added in recommended amounts to food. However, this label does not apply to inhalational safety, and short term occupational exposures to propylene glycol caused irritation and either mild or no objective effects on pulmonary function, suggesting that propylene glycol may act as a sensory irritant.^116^ ^118^ ^132^ ^133^ Propylene glycol activated TRPV1 and TRPA1, two irritant receptors expressed in sensory nerves innervating the airways.^134^ ^135^ These receptors promote asthmatic inflammation and airway hyper-reactivity in asthma models.^136^ MUC5AC protein concentrations were increased in the lungs of chronic vapers.^57^ Propylene glycol/vegetable glycerin, and not nicotine, increased mucin expression after vaping in primary airway epithelia.^57^ Additional studies into their effects on pulmonary and immune cells are needed. Propylene glycol and vegetable glycerin can enter cells through several aquaporins including AQP3, which is expressed in the lung, suggesting that they may exert some effects intracellularly,^137^ ^138^ ^139^ and vegetable glycerin can affect biologic membranes.^140^ Consistent with this, propylene glycol and vegetable glycerin decreased membrane fluidity in airway epithelia.^55^ Decreases in membrane fluidity may affect endocytosis (including phagocytosis, a specialized form of endocytosis), exocytosis, and plasma membrane protein-protein interactions. “The dose and the route make the poison” is a founding principle of toxicology, and high doses of inhaled propylene glycol/vegetable glycerin, which may occur during chronic vaping, likely contribute to the nicotine independent effects that have been described. Therefore, the safety of propylene glycol and vegetable glycerin at levels inhaled by e-cigarette users remains uncertain.

### Nicotine

Nicotinic acetylcholine receptors (nAChR) are ligand gated ion channels expressed in the airways.^141^ ^142^ ^143^ ^144^ nAChR activation increases cytosolic Ca^2+^ levels and can inhibit CFTR in airway epithelia.^145^ Nicotine can also affect alveolar macrophages.^116^ Protease and interleukin 8 secretion from neutrophils is also independent of nicotine. Furthermore, inhaled nicotine increases elastase gene expression in neutrophils.^146^ ^147^ However, the effects of nicotine can be extensive and varied, as described elsewhere.^148^

nAChRs can regulate cell proliferation and inhibit apoptosis,^149^ and uncontrolled cell proliferation is a hallmark of cancer. In genome-wide association studies, α3, α5, and β4 nAChR were associated with lung cancer.^150^ ^151^ ^152^ ^153^ Additionally, differential nAChR expression profiles between non-smokers and smokers with non-small cell lung cancer were observed.^154^ Whether chronic activation of nAChR to nicotine via e-cigarettes can cause lung cancer is unknown, but the role of nAChR α7 in contributing to non-small cell lung cancer by altering cell proliferation and apoptotic resistance has been reported.^154^ ^155^ Nicotine contributes to vascular endothelial dysfunction by increasing leakiness.^156^ Furthermore, exposure to nicotine, but not propylene glycol/vegetable glycerin, increased arterial stiffness and adversely affected the microcirculation,^157^ suggesting that nicotine delivered by e-cigarettes may be a risk factor for cardiovascular disease. Nicotine exposure from e-cigarettes will likely have pharmacologic effects in any organ where nAChR are expressed. Thus, e-cigarette use may affect inflammation in the airways that could alter susceptibility to infection and/or increase the risk of developing COPD or lung cancer.

### Flavors

E-liquids contain many flavors including aldehydes (vanillin, vanilla; benzaldehyde, berry/fruit; cinnamaldehyde, cinnamon; damascenone, tobacco), benzyl alcohol, terpenes (linalool, flowery; farnesol, apple), pyrazines (coffee, chocolate), menthol, menthone and other minty compounds, and sweet flavors including ethyl maltol. The combination of these and many other chemicals gives rise to the thousands of marketed flavored e-liquids. Many flavors are used as food additives and scents in cosmetics. However, their safety in the lung, at levels inhaled by e-cigarette users, is uncertain.^158^ Whereas occupational exposures to these flavors are regulated, concentrations in e-cigarettes are not.^159^ This is of concern as aldehyde flavors can be hazardous at elevated concentrations. However, as with nicotine, the flavor concentrations seen in the lungs during vaping are unknown.

An in vitro study assessed about 150 e-liquids and found a positive correlation between the number of flavors in an e-liquid and its in vitro toxicity.^117^ It also found that the concentrations of vanillin and cinnamaldehyde in different e-liquids correlated with overall toxicity. Concentrations of cinnamaldehyde in e-liquids can exceed 1 M (molar), and cinnamaldehyde flavored e-cigarette aerosols caused cytotoxicity and ciliary dysfunction in epithelia and inflammation in vivo.^115^ ^160^ ^161^ ^162^ ^163^ Great concern has been expressed about the presence of the buttery flavor diacetyl in e-liquids, owing to its known pulmonary toxicity and propensity for causing bronchiolitis obliterans.^158^ ^159^ ^163^ ^164^ Intriguingly, within hours after mixing, aldehyde flavors can undergo chemical reactions with propylene glycol/vegetable glycerin, forming acetal compounds. These compounds, which are stable in aqueous environments at physiologic pH, as an aerosol can activate irritant receptors.^165^ Thus, e-liquids are much more complex than initially thought and are chemically unstable, forming compounds with novel respiratory toxicological effects.

### Degradation products (eg, aldehydes)

Initial studies reported that formaldehyde was formed during the vaping process under high heat conditions.^166^ Although some of the more recent e-cigarette devices limit temperature in an attempt to minimize this, multiple reports have documented the formation of acetaldehyde, acrolein, diacetyl, and formaldehyde under a wide range of conditions.^167^ ^168^ Intermediate products including glycidol and acetol have also been detected, suggesting that these carbonyls are likely produced from heated propylene glycol/vegetable glycerin through oxidation.^169^ Glycidol, an epoxide, is an irritant and tightly controlled known carcinogen, and acrolein is a potent irritant and the major non-cancer hazard in tobacco smoke,^170^ ^171^ suggesting that these degradation products are relevant to lung health.

## Effects on population health

In the US, the Food and Drug Administration (FDA) is required to consider the net effects of regulatory policy on population health. Although a full review of this topic is beyond the scope of this review, recent assessments have come to different conclusions about whether possible harm reduction benefits of substituting e-cigarette for cigarettes could outweigh adverse effects of e-cigarettes among never smokers, ex-smokers continuing to use e-cigarettes, and dual users of cigarettes and e-cigarettes.^172^ ^173^ These inconsistent conclusions reflect uncertainty about the long term health effects of e-cigarettes, their effectiveness as smoking cessation agents, and their effect on children. A modest increase in risk of e-cigarette attributable respiratory diseases such as lung cancer or COPD, or cardiovascular disease, could markedly shift the net evidence base on population effects to support more restrictive regulatory policy on e-cigarettes. Effects of other little studied outcomes of e-cigarette exposure, including in utero exposure to maternal e-cigarette use and secondhand exposure to e-cigarette aerosol in bystanders, could also shift the assessment of respiratory and other population effects.^174^ ^175^ In the US, the assessment of effects may change on the basis of the large increase in use of e-cigarettes in school age young people in 2018, which drove an increase in overall use of any tobacco product, largely erasing recent progress in reducing use in young people.^176^ What has also become clear is that e-cigarette use among young people is a strong risk factor for subsequent initiation of combustible cigarettes.^4^

In large multi-country studies, and in a comprehensive review and meta-analysis of observational cohort studies, e-cigarette use was associated with reduced cigarette cessation,^177^ ^178^ although these associations may vary by pattern of use and type of device.^179^ In contrast, the evidence from randomized trials that e-cigarettes are effective for smoking cessation is limited and mixed.^40^ ^180^ ^181^ One recent randomized trial showed that e-cigarettes were superior to nicotine patches for smoking cessation at one year (18% compared with 9.9%).^40^ Although these results were promising, an accompanying editorial noted that the rate of quitting in the e-cigarette group was not superior to rates associated with FDA approved pharmacotherapy in other studies.^182^ In addition, 80% of the e-cigarette group was still using e-cigarettes at one year, compared with 9% of the nicotine replacement therapy group, thus subjecting the e-cigarette group to sustained risks, including dual e-cigarette and cigarette use.

### Differences in regulatory approaches

Given uncertainties in health effects and harm reduction potential, different regulatory approaches have been adopted in different countries, with the UK at one extreme promoting the use of e-cigarettes for harm reduction,^183^ and the US, the European Union, and other countries on a spectrum from harm reduction to a principle of precaution. The position of respiratory and other academic and public health societies has generally emphasized a precautionary approach, with the notable exception of the UK’s Royal College of Physicians.^184^ ^185^ ^186^ ^187^ ^188^ ^189^ For example, a recent European Respiratory Society panel concluded that as the chronic effects of e-cigarette use are unknown, no evidence shows that they are safer than other tobacco products and that, on the basis of current knowledge, negative health effects cannot be excluded.

## Challenges and guidelines for clinicians

Although several large and influential organizations have published official statements and guidelines pertaining to the use of e-cigarettes,^4^ ^183^ ^190^ device technology and corresponding patterns of use are rapidly changing, and research continues to advance our understanding of health effects. Therefore, these recommendations can quickly become dated (box 1). For example, the addictive potential of nicotine salts may have contributed to the dramatic recent increase in 2018 in vaping among young people in the US, fueled by Juul. However, this addictive potential may be more satisfying to smokers who are switching to e-cigarettes.^19^ We note, however, that whereas Juul are currently sold with 59 mg/mL of nicotine salt in the US, they can be sold only with 18 mg/mL in the UK. Thus, we speculate that this may influence their relative popularity on either side of the Atlantic and/or influence their addictiveness versus success in smoking cessation.

Box 1 **Challenges in the study of novel tobacco products/e-cigarettes**

- Rapid introduction of new devices (Heat-Not-Burn/IQOS)
- Minimal information from vendors about e-liquid content
- Rapid evolution of existing e-cigarette technology—devices may be obsolete by the time a study is finished and published
- Lack of a standard “e-cigarette liquid/device” vis à vis “Kentucky Research cigarettes”
- Lack of standardization for e-cigarette aerosol generation and exposure
- Variability in operating parameters for devices (power, ambient conditions)
- Dual use of combustible cigarettes with other tobacco products including e-cigarettes, hookah, and marijuana
- Different devices across different countries and regulatory domains—for example, Juul contains 59 mg/mL nicotine in the US and 18 mg/mL in the UK
- Ethical considerations make study of addictive and potentially harmful liquids/devices in never-smokers challenging
- Many of the important outcomes related to chronic toxicity (for example, chronic obstructive pulmonary disease) take many years to develop, so the true effects may not be known for decades
- The effect on vulnerable populations (such as people with asthma, chronic obstructive pulmonary disease, and/or lung cancer) may differ from the effects seen in young healthy normal people

**Fig 2:**
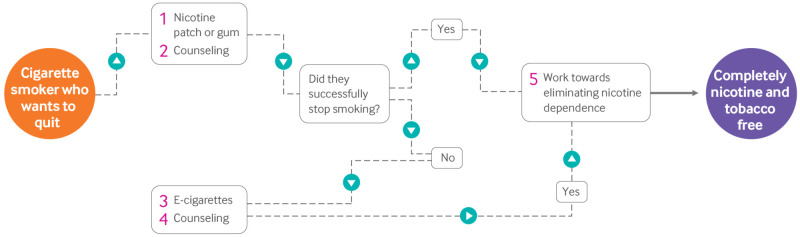
Flowchart outlining proposed smoking cessation regimen that espouses counseling and nicotine cessation. Given the potential health risks associated with vaping, tobacco smokers looking to quit should first try nicotine patch or gum along with counseling (1, 2). If this approach fails, e-cigarettes could be used as a second attempt (3, 4). Given that nicotine is not risk-free, attempts should then be made to end nicotine dependence (5). As nicotine levels in e-liquids differ globally, the use of e-cigarettes as a smoking/nicotine cessation device may be more effective in countries with lower nicotine levels

The uncertainty about the health risks of e-cigarettes and their efficacy as smoking cessation agents poses a challenge to clinicians, as smokers are using e-cigarettes in attempts to quit smoking and they look to their physicians for guidance. The use of evidence based pharmacotherapy and nicotine patches that are safe and effective in reducing the dependence on nicotine in cigarettes, combined with counseling, is the only approach for which little therapeutic or health uncertainty exists (fig 2).^191^ A supportive environment including counseling has been key to the success of this approach, and smokers should be encouraged to take advantage of complementary community and therapeutic resources. Smokers and ex-smokers using e-cigarettes should be provided with clear information on the uncertainties about health risks and harm reduction and encouraged to participate in complementary counseling using established approaches with a goal of quitting all tobacco products and ultimately reducing nicotine dependency as soon as possible. Smokers should, in particular, be cautioned about the hazards of dual cigarette and e-cigarette use, which may impede quit attempts, and the recently discovered potential risks of switching to e-cigarettes.

Pediatricians are faced with an epidemic of e-cigarette use for which there is arguably no benefit and potentially substantial, albeit uncertain, health risk.^192^ Communication of this risk to young people and parents is a key service that pediatricians can provide. For young people, prevention is key, and clinicians should recommend evidence based treatment for those using tobacco products.^5^ Because the community and policy makers look to physicians for information on children’s health, the pediatrician has a unique opportunity to promote action, including enforcement of age, sales, and marketing limitations, raising the legal age limit for tobacco use to 21, and innovative regulation such as banning flavored tobacco products and other public health action.

## How to perform long term toxicology studies to assess the effects of e-liquids?

Even though e-cigarettes contain strongly psychoactive substances (nicotine or nicotine salt), they do not require rigorous testing before being marketed. In contrast, for a new pharmaceutical product to reach the market requires a well defined approach that includes preclinical toxicology in animal models followed by robust clinical trials.^193^ We propose that e-cigarettes be similarly regulated and evaluated in a well defined and transparent series of preclinical, time appropriate animal models. Although conducting preclinical toxicological studies in animals is fairly straightforward, doing “clinical trial” type studies in humans leads to interesting ethical considerations. Administering e-cigarettes to healthy non-smokers would be unethical. However, given that many current smokers are in the process of developing lung pathology and e-cigarette users’ lungs also seem to be undergoing changes, will conducting clinical trials in current smokers be appropriately informative? Using rigorous and transparent preclinical studies to inform both vendors and the general public as to the relative effects of different e-liquids by using the same approach used for potential therapeutics would be a step forward. Certainly, given that vaping is now a multibillion dollar industry, the e-cigarette companies would seem to have the means to support these studies.

## Conclusions

We reiterate that, to date, no long term vaping toxicological/safety studies have been done in humans; without these data, saying with certainty that e-cigarettes are safer than combustible cigarettes is impossible. Box 1 outlines the challenges facing the field. Given the survey data showing increased symptoms of respiratory disease and the many lines of human, animal, and in vitro experimental evidence that e-cigarette aerosol can negatively affect multiple aspects of lung cellular and organ physiology and immune function, e-cigarettes will likely prove to have at least some pulmonary toxicity with chronic and possibly even short term use. Several important principles will determine how lung disease manifests and how severely: as with smokers, vapers are likely to have variable susceptibility to lung injury, influenced by many interacting genetic and environmental factors; certain variations of e-cigarette technology (atomizer construction, coil power, nicotine exposure, and flavorants) will prove more harmful than others; dual use with combustible cigarettes, the dominant adult use pattern, may potentiate toxicity; a critical factor will be the extent to which vaping alters the susceptibility to and trajectory of bacterial and viral lung infections; and the continued rapid technological evolution of these devices may mitigate or potentiate particular toxicities.

**Research questions**

- Does inhaled nicotine cause direct pulmonary toxicity?
- What is the significance of lipid laden macrophages in e-cigarette associated lung disease?
- Do e-cigarettes have detrimental effects on adolescent lung development?
- What is the effect of vaping on vulnerable populations (those with pre-existing conditions such as asthma or chronic obstructive pulmonary disease)?
- Does e-cigarette use lead to immunosuppression?

## Notes

Provenance and peer review: Commissioned; externally peer reviewed.

Patient involvement: No patients were involved in the drafting or review of this manuscript.
